# Religious identity cues increase vaccination intentions and trust in medical experts among American Christians

**DOI:** 10.1073/pnas.2106481118

**Published:** 2021-11-18

**Authors:** James Chu, Sophia L. Pink, Robb Willer

**Affiliations:** ^a^Department of Sociology, Columbia University, New York, NY 10027;; ^b^Department of Sociology, Stanford University, Stanford, CA 94305

**Keywords:** vaccines, COVID-19, religiosity, common identity cues

## Abstract

Containing the COVID-19 pandemic in the United States requires mobilizing a large majority of the mass public to vaccinate, but many Americans are hesitant or opposed to vaccination. A significant predictor of vaccine attitudes in the United States is religiosity, with more-religious individuals expressing more distrust in science and being less likely to get vaccinated. Here, we test whether explicit cues of common religious identity can help medical experts build trust and increase vaccination intentions. In a preregistered survey experiment conducted with a sample of unvaccinated American Christians (*n* = 1,765), we presented participants with a vaccine endorsement from a prominent medical expert (NIH Director Francis Collins) and a short essay about doctors’ and scientists’ endorsement of the vaccines. In the common religious identity condition, these materials also highlighted the religious identity of Collins and many medical experts. Unvaccinated Christians in the common identity condition expressed higher trust in medical experts, greater intentions to vaccinate, and greater intentions to promote vaccination to friends and family than those who did not see the common identity cue. These effects were moderated by religiosity, with the strongest effects observed among the most religious participants, and statistically mediated by heightened perceptions of shared values with the medical expert endorsing the vaccine. These findings demonstrate the efficacy of common identity cues for promoting vaccination in a vaccine-hesitant subpopulation. More generally, the results illustrate how trust in science can be built through the invocation of common group identities, even identities often assumed to be in tension with science.

The availability of effective vaccines against severe acute respiratory syndrome coronavirus 2 offers a potential resolution to the COVID-19 pandemic, the most serious global health crisis in over a century. Motivating the high levels of vaccination needed for containment, however, has proven difficult in the United States, with 37.7% of eligible Americans still not fully vaccinated as of September 7, 2021 (1). While research on vaccine hesitancy has focused on political identity (2), religious identity is also a strong predictor of general vaccine hesitancy among Americans, even controlling for political orientation (3). Religious identity is specifically related to COVID-19 vaccination intentions, with American Protestants, for example, being especially skeptical of the vaccines (4). A likely contributor to the link between religious identity and vaccine skepticism is the tendency for more-religious people to put less trust in scientific and medical experts (5). While many assume that conflict between religion and science is rooted in divergent views of how claims about the natural world should be made, for example, regarding human origins, prior work finds that lack of trust in science among religious people is rooted more in perceptions that scientists and religious people hold divergent values (6).

To address these problems, we test whether invocations of common religious identity can increase trust in, and the influence of, medical experts. We also explore the effects of common identity invocations on perceptions of shared values. Because religious identities are diverse in the United States, we focus our study on a single religious group, American Christians. Among religious groups in the United States, Christians both are the largest group and have low COVID-19 vaccination rates, making them a population of unique interest for containing the pandemic (7).

Research on religion and science suggests that invoking common religious identities is likely to improve the credibility of scientists (8, 9). Additionally, research on social identity theory finds that highlighting common, valued identities can foster trust, cooperation, and alignment on important issues (10). For example, studies find that individuals are more persuaded by others with whom they share a meaningful group identity, such as political party identification (11), or even a seemingly trivial commonality, like a common birthplace or birthday (12).

Here we test whether this prior work applies in the current context, where trust in medical experts and the COVID-19 vaccines are deeply divided in the American public. Notably, organizations like the US Department of Health and Human Services have recruited religious leaders to persuade Americans to receive COVID-19 vaccinations since spring 2021 (13). While religious leaders may be persuasive, they typically lack expertise on transmission dynamics and vaccine safety and efficacy. We propose a different, although complementary, approach. We predict that medical experts can build trust and motivate vaccination among religious groups by invoking a common religious identity.

We test this claim in a preregistered survey experiment. In May and June 2021, we recruited 1,765 online participants who reported being unvaccinated, Christian, and at least moderately religious. The experimental manipulation was highly controlled, with minimal differences in stimuli between conditions. Participants randomly assigned to the control condition were shown a biography of NIH Director Francis Collins. They then viewed a video where Collins responded to questions about the efficacy and safety of the COVID-19 vaccines, and they read a short essay about how medical experts endorse vaccinations. Participants assigned to the common identity condition read the same content, but the content also highlighted the Christian identity of medical experts. The biography identified Collins as a Christian, and Collins began the video declaring his “trust in Jesus as the source of all truth.” While Collins' background is Protestant, and this statement is Protestant in tone, we expected the common identity condition would be persuasive to Christians in general because Protestants are likely perceived as having shared values with all Christians, and it would effectively contradict the assumption that medical experts are secular. Participants then read the same essay about how medical experts endorsed vaccination, with the addition of statistics showing that many medical experts are people of faith (Fig. 1). Finally, we measured participants’ vaccination intentions, their intentions to encourage family and friends to vaccinate, and their trust in the medical expert. Because prior work explains distrust of medical experts in religious communities in terms of perceived value differences, we also measured participants’ perceptions of shared values with medical experts.

**Fig. 1.**
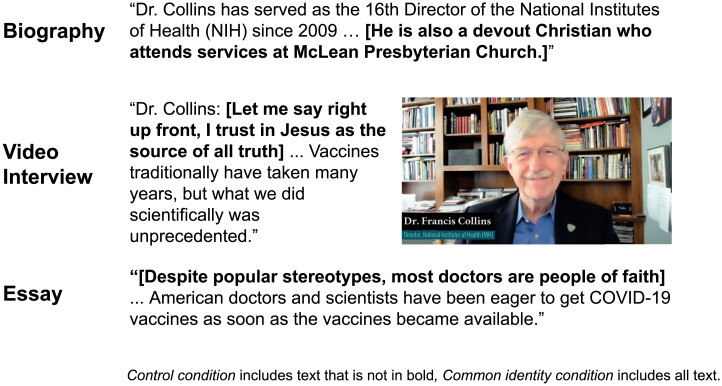
Excerpts from biography, video, and essay in control and common identity condition.

## Results

**Fig. 2.**
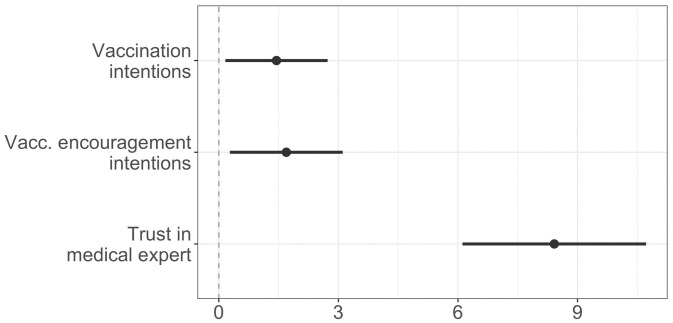
Estimated effects of common identity on vaccination intentions, willingness to encourage others to get a vaccine, and trust in the medical expert. All estimates are on a 100-point scale.

Fig. 2 shows the estimated effects of the common identity condition, relative to the control condition. The estimates are based on models controlling for demographic characteristics (age, gender, race, education, and income) and vaccination intentions reported pretreatment.

In a preregistered analysis, we found that participants in the common identity condition reported greater vaccination intentions than participants in the control group (β = 1.45, CI = [0.16, 2.73], *P* = 0.03). Participants in the common identity condition also reported increased willingness to encourage others to get vaccinated (β = 1.69, CI = [0.27, 3.11], *P* = 0.02). Additionally, participants in the common identity condition perceived the medical expert as more trustworthy (β = 8.42, CI = [6.11, 10.72], *P* < 0.001). Participants in the common identity condition were also more likely to believe that the medical expert shared their values (β = 14.2, CI = [12.1, 16.3], *P* < 0.001).

The effects on vaccination intentions, willingness to encourage others to get vaccinated, and trust in the medical expert were significantly moderated by religiosity. We found larger effects among people who reported, premanipulation, that they were more religious (interaction terms: β_vacc_intentions_ = 1.20, *P* = 0.05; β_encourage_others_ = 1.41, *P* = 0.03; β_trust_ = 2.86, *P* = 0.008). Statistical mediation analyses suggest that shared values with the medical expert mediate the effects of common identity on vaccination intentions (indirect effect = 3.37, *P* < 0.001), encouragement intentions (indirect effect = 5.37, *P* < 0.001), and trust in the medical expert (indirect effect = 12.25, *P* < 0.001).

## Discussion

Hearing about the common religious identity of medical experts led unvaccinated Christians to intend to receive one of the COVID-19 vaccines, encourage others to vaccinate, and report greater trust for medical experts. Notably, these results were more pronounced among more-religious Christians, a subpopulation high in vaccine hesitancy. Highlighting common identity increased participants’ perceptions that the medical expert shared their values, and results of a statistical mediation analysis suggested that shared values drove the treatment effects on vaccination intentions, vaccination encouragement intentions, and trust. These findings have three important implications.

First, our results extend prior research on the role of trusted sources in encouraging health behaviors. While trusted religious sources have been shown to be helpful during public health crises (14), we demonstrate the efficacy of highlighting the religious identities of many medical experts. This is uniquely valuable because medical experts are typically the best informed about scientific evidence and can communicate public health information more accurately than clergy. These results suggest vaccine advocacy efforts by organizations seeking to connect scientists who are Christians with Christians in the general public (e.g., BioLogos, Christians and the Vaccine, or Science for the Church) may be effective. Future work should test the generalizability of these claims to religious identities beyond Christianity and compare the magnitude of effects relative to cues for other identity dimensions, such as racial or ethnic identities.

Second, we show how religious and scientific identities can be complementary, and our findings support claims about the malleability of boundaries between science and religion (9). Science is often perceived to be in tension with religion, and, if so, invoking the religious identities of medical experts could have eroded their scientific authority and persuasiveness. Our results show, however, that invoking common religious identities with medical experts led to increases in vaccination intentions and willingness to encourage vaccination, even in a highly vaccine-hesitant population.

Third, we clarify the mechanisms driving gaps in vaccination observed in current public health data. Our findings suggest that Christians are less likely to support vaccinations, in part, because they do not perceive overlapping identities and shared values with medical experts. Although 76% of doctors report believing in God (15), awareness of this fact is likely low. Thus, in addition to efforts to recruit religious authorities to advocate for vaccinations, efforts to make salient the religious background of medical experts could help increase trust in medical experts and the rate of vaccinations in the United States.

## Methods

In May and June 2021, we recruited 1,765 participants via Lucid, CloudResearch, and Amazon Mechanical Turk for an online survey. Based on preregistered procedures, we included participants who passed a video attention check and a written attention check, indicated they had not received any dose of a COVID-19 vaccine, self-identified as Christian, and indicated at least four on a seven-point scale when asked how religious they were. Premanipulation, participants were asked to indicate how likely they would be to get a COVID-19 vaccine. Participants were then randomly assigned to the common identity condition or the control condition, using block randomization based on pretreatment vaccination intentions (*SI Appendix*). Afterward, participants were asked about their vaccination intentions, willingness to encourage others to get a vaccine, trust in the doctor in the video, and belief that the doctor in the video shared the same values as them, and additional, exploratory measures. We did not include survey weights in our analyses, because we lack data on the demographics of unvaccinated Christians, and we could not preregister a weighting scheme for this population whose demographic composition was shifting. See *SI Appendix* for the full text of stimuli and survey items. Research was approved by the Stanford University Institutional Review Board. All subjects provided informed consent.

## Supplementary Material

Supplementary File

## Data Availability

Study materials, analysis code, and data are available at Open Science Framework (https://osf.io/2uk5d/).

## References

[1] Centers for Disease Control and Prevention, “COVID-19 vaccinations in the United States.” CDC Data Tracker. https://covid.cdc.gov/covid-data-tracker/#vaccinations. Accessed 8 September 2021.

[2] S. L. Pink, J. Chu, J. N. Druckman, D. G. Rand, R. Willer, Elite party cues increase vaccination intentions among Republicans. Proc. Natl. Acad. Sci*. U.S.A.* 118, e2106559118 (2021) 34312254 10.1073/pnas.2106559118 PMC8364165

[3] B. T. Rutjens, R. M. Sutton, R. van der Lee, Not all skepticism is equal: Exploring the ideological antecedents of science acceptance and rejection. Pers. Soc. Psychol. Bull. 44, 384–405 (2018). 29191107 10.1177/0146167217741314 PMC5810918

[4] C. Funk, J. Gramlich, “10 facts about Americans and coronavirus vaccines.” Pew Research Center. https://www.pewresearch.org/fact-tank/2021/03/23/10-facts-about-americans-and-coronavirus-vaccines/. Accessed 20 September 2021.

[5] E. Chan, Are the religious suspicious of science? Investigating religiosity, religious context, and orientations towards science. Public Underst. Sci. 27, 967–984 (2018). 29874969 10.1177/0963662518781231

[6] J. Evans, Morals Not Knowledge: Recasting the Contemporary U.S. Conflict between Religion and Science (University of California Press, 2018).

[7] Pew Research Center, “Religious Landscape Survey.” https://www.pewforum.org/religious-landscape-study/. Accessed 20 September 2021.

[8] E. H. Ecklund, Science vs. Religion: What Scientists Really Think (Oxford University Press, 2010).

[9] C. P. Scheitle, E. H. Ecklund, The influence of science popularizers on the public’s view of religion and science: An experimental assessment. Public Underst. Sci. 26, 25–39 (2017). 26055875 10.1177/0963662515588432

[10] N. Ellemers, S. A. Haslam, “Social identity theory” in Handbook of Theories of Social Psychology, A. W. Kruglanski, E. T. Higgins, P. van Lange, Eds. (Sage, Chicago, Il, 2011), vol. 2, pp. 379–398.

[11] J. Marks, E. Copland, E. Loh, C. R. Sunstein, T. Sharot, Epistemic spillovers: Learning others’ political views reduces the ability to assess and use their expertise in nonpolitical domains. Cognition 188, 74–84 (2019). 30342868 10.1016/j.cognition.2018.10.003 PMC6522687

[12] L. Jiang, J. Hoegg, D.W. Dahl, A. Chattopadhyay, The persuasive role of incidental similarity on attitudes and purchase intentions in a sales context. *J.* Consum. Res. 36, 778–791 (2010).

[13] Z. Miller, “Biden launches community corps to boost COVID vaccinations.” *The Associated Press*, 1 April 2021. https://apnews.com/article/public-health-television-media-celebrity-social-media-4040be9cc79b78f5fea35ed37a9d4db1. Accessed 20 September 2021.

[14] C. Greyling , Lessons from the faith-driven response to the West Africa Ebola epidemic. Rev. Faith Int. Aff. 14, 118–123 (2016).

[15] F. A. Curlin, J. D. Lantos, C. J. Roach, S. A. Sellergren, M. H. Chin, Religious characteristics of U.S. physicians: A national survey. J. Gen. Intern. Med. 20, 629–634 (2005). 16050858 10.1111/j.1525-1497.2005.0119.x PMC1490160

