# Supplementary material for: Religious identity cues increase vaccination intentions and trust in medical experts among American Christians
Source: Proc Natl Acad Sci U S A. 2021 Nov 18;118(49):e2106481118. doi: 10.1073/pnas.2106481118 (PMC8670469; doi:10.1073/pnas.2106481118)
Supplement: Supplementary File [file pnas.2106481118.sapp.pdf]

Supplementary Information for

Religious Identity Cues Increase Vaccination Intentions and Trust in Medical Experts among American Christians

Treatment and control condition materials

Source for Interview Content and Video:
Chang, Curtis and Kris Carter. [Redeeming Babel] (2021, April 5). A Conversation with Dr. Francis Collins [Video]. YouTube. https://www.youtube.com/watch?v=kP-LyjY37t8&t=2s

Biography of medical expert: Common identity condition

On the next page, you'll watch an interview with Francis S. Collins, M.D, Ph.D.

Dr. Collins has served as the 16th Director of the National Institutes of Health (NIH) since 2009. He is also a devout Christian who attends services at McLean Presbyterian Church in Northern Virginia. After converting to Christianity from atheism in his 20s, Dr. Collins has worked to connect science and faith through his work. He is author of the best-selling book, "The Language of God: A Scientist Presents Evidence for Belief."

Dr. Collins is a physician-geneticist noted for his landmark discoveries of disease genes and his leadership of the international Human Genome Project. He served as director of the National Human Genome Research Institute at NIH from 1993-2008. He also received the Templeton Prize, which honors people who use science to explore questions of spirituality.

Along with religious leaders like theologian N.T. Wright and former pastor Tim Keller, as well as leaders from the scientific community, Dr. Collins has led efforts to promote the safety and efficacy of COVID-19 vaccines.

Biography of medical expert: Control condition

On the next page, you'll watch an interview with Francis S. Collins, M.D, Ph.D.

Dr. Collins has served as the 16th Director of the National Institutes of Health (NIH) since 2009. Dr. Collins is a physician-geneticist noted for his landmark discoveries of disease genes and his leadership of the international Human Genome Project. He served as director of the National Human Genome Research Institute at NIH from 1993-2008. He has also authored several best-selling books.

Along with other public health leaders and doctors, Dr. Collins has led efforts to promote the safety and efficacy of COVID-19 vaccines.

Interview: Common identity condition

Transcript of the video interview with Dr. Francis Collins below. Video content was sourced from Christians and the Vaccine, a group producing content to persuade Christian audiences to get vaccinated:

DR. CHANG: Dr. Collins, welcome and thanks for joining us.

DR. COLLINS: Thanks, Curtis. I'm glad to be with you, and I'm glad to talk about trust and let me say right up front, I trust in Jesus as the source of all truth and I'm sure that we would agree that's our best foundation.

PROF CHANG: Absolutely, amen. So let's get into it. The speed that these vaccines were developed, it makes me nervous. Don't vaccines usually take many years to develop? Was this rushed and corners cut?

DR. COLLINS: It is the case that vaccines traditionally have taken many years, but this was a crisis with millions of people's lives at risk. So what we did scientifically was unprecedented. We basically identified all of the downtime that happens when a vaccine is being developed and tried to eliminate that and organize all of those steps so that they happen seamlessly one after the other. Always ready, of course, to abandon a vaccine that failed to meet the highest standards of safety and efficacy. And the good news was we didn't have to abandon them because the results were even better than I could have really dared to hope for. And so in just 11 months with probably the most rigorous analysis that's ever been done of a new vaccine, we were able to see that they actually were safe and 95% effective in preventing disease but no corners were cut. This was done with the most extreme sort of perspective about scientific rigor that could possibly have been imagined in such a short period of time. So people can trust that this was done right.

PROF CHANG: What about long-term side effects? These vaccines are so new. How do we know there isn't something horrible lurking down the road?

DR. COLLINS: It is true that we've only known about this virus for a little over a year. And so there's a lot that we don't know about the disease itself. And of course we can't say whether there might be some very long-term side effects of the vaccines. But the facts are that if you look at every other vaccine that's ever been developed, if you're going to see serious side effects, those generally become clear in the first couple of months after people get injected. That's why in the trials, FDA was not going to accept any of these vaccines until two months had passed for the people who were involved in those large scale trials to make sure that they weren't seeing unexpected side effects and they didn't. So that is about the most reassurance one can get in this

situation. When you consider the risks of the disease, that still seems like the risk of any uncertain long-term side effect is very small by comparison.

PROF CHANG: Dr. Collins, you're well-known as a follower of Jesus but some people think that faith in Jesus and faith in science are in opposition, can you speak to that?

DR. COLLINS: I would love to speak to that because it is truly unfortunate that there is this sense that science and faith are in conflict. I became a Christian at age 27. People said to me, 'cause I was always already on a path to be a physician and a scientist that my head was going to explode. It never happened. I see these as wonderfully complementary and harmonious. Science allows us to investigate God's creation and being a scientist is also an opportunity to be a person who worships what God has done by creating nature around us. You have to think about what kind of question you're asking whether science or faith is the way to approach it. But I think I agree with Francis Bacon who said we were given two books, the book of God's words, the Bible, which I study every morning and the book of God's works, this amazing complex, beautiful creation which we also have the chance through science to understand and to use that even for the purpose of healing which I believe also is part of God's intention. There's no conflict here.

Interview: Control condition

Transcript of the video interview with Dr. Francis Collins below:

PROF CHANG: The speed that these vaccines were developed, it makes me nervous. Don't vaccines usually take many years to develop? Was this rushed and corners cut?

DR. COLLINS: It is the case that vaccines traditionally have taken many years, but this was a crisis with millions of people's lives at risk. So what we did scientifically was unprecedented. We basically identified all of the downtime that happens when a vaccine is being developed and tried to eliminate that and organize all of those steps so that they happen seamlessly one after the other. Always ready, of course, to abandon a vaccine that failed to meet the highest standards of safety and efficacy. And the good news was we didn't have to abandon them because the results were even better than I could have really dared to hope for. And so in just 11 months with probably the most rigorous analysis that's ever been done of a new vaccine, we were able to see that they actually were safe and 95% effective in preventing disease but no corners were cut. This was done with the most extreme sort of perspective about scientific rigor that could possibly have been imagined in such a short period of time. So people can trust that this was done right.

PROF CHANG: What about long-term side effects? These vaccines are so new. How do we know there isn't something horrible lurking down the road?

DR. COLLINS: It is true that we've only known about this virus for a little over a year. And so there's a lot that we don't know about the disease itself. And of course we can't say whether there might be some very long-term side effects of the vaccines. But the facts are that if you look at every other vaccine that's ever been developed, if you're going to see serious side effects, those generally become clear in the first couple of months after people get injected. That's why in the trials, FDA was not going to accept any of these vaccines until two months had passed for the people who were involved in those large scale trials to make sure that they weren't seeing unexpected side effects and they didn't. So that is about the most reassurance one can get in this situation. When you consider the risks of the disease, that still seems like the risk of any uncertain long-term side effect is very small by comparison.

Essay: Common identity condition

Most Doctors and Scientists are Religious

Most people do not realize that the majority of American doctors and scientists are religious. In one recent survey of 2,000 medical doctors in the United States, 89% identified as religious, 76% said that they believe in God, and more than 60% identified as Christian. This shows that, despite popular stereotypes, most doctors are people of faith, and the majority are Christians.

The same is true for scientists. A survey of 1,386 scientists conducted by researchers at Rice University found that 85% were raised with religious traditions. In one survey of scientists, 85% reported that they believed that science and religion can coexist without contradicting one another. Thus, while many view science and religion as at odds with one another, survey research shows that most scientists do not see fundamental contradictions between science and religion, and most are people of faith.

American Doctors and Scientists Encourage Adults to Get Vaccinated

American doctors and scientists have been eager to get COVID-19 vaccines as soon as the vaccines became available. A survey of 3,000 physicians found that, as of February 2020, 95% of doctors had already received or planned to receive a COVID-19 vaccine.

Doctors and scientists are advocating for all adults to get a COVID-19 vaccine. Dr. Susan R. Bailey, President of the American Medical Association, explained, “Vaccinations are safe, effective, prevent illness, and save lives.” Similarly, Dr. Ernest J. Grant, President of the American Nurses Associated, emphasized that “vaccination will help protect us from this deadly virus.” Dr. David Schultz, who runs an independent practice in North Carolina, said, “I think that every physician is just really relieved and energized to get people vaccinated.”

Sources:
Bernard, Rebekah (2021). Physicians highly accepting of COVID-19 vaccine. Medical Economics.
Ecklund, E. H., & Park, J. Z. (2009). Conflict between religion and science among academic scientists?. Journal for the Scientific Study of Religion, 48(2), 276-292.
Ecklund, E. H., Park, J. Z., & Sorrell, K. L. (2011). Scientists negotiate boundaries between religion and science. Journal for the Scientific Study of Religion, 50(3), 552-569.
Curlin, F. A., Lantos, J. D., Roach, C. J., Sellergren, S. A., & Chin, M. H. (2005). Religious characteristics of US physicians. Journal of General Internal Medicine, 20(7), 629-634.

Essay: Control condition

American Doctors and Scientists Encourage Adults to Get Vaccinated

American doctors and scientists have been eager to get COVID-19 vaccines as soon as the vaccines became available. A survey of 3,000 physicians found that, as of February 2020, 95% of doctors had already received or planned to receive a COVID-19 vaccine.

Doctors and scientists are advocating for all adults to get a COVID-19 vaccine. Dr. Susan R. Bailey, President of the American Medical Association, explained, “Vaccinations are safe, effective, prevent illness, and save lives.” Similarly, Dr. Ernest J. Grant, President of the American Nurses Associated, emphasized that “vaccination will help protect us from this deadly virus.” Dr. David Schultz, who runs an independent practice in North Carolina, said, “I think that every physician is just really relieved and energized to get people vaccinated.”

Sources: Bernard, Rebekah (2021). Physicians highly accepting of COVID-19 vaccine. Medical Economics.

Participants

We recruited participants from Lucid (79% of the sample) and Cloud Research / Mechanical Turk (21% of the sample). In order to qualify for the study, participants had to: a) pass two brief attention checks, one asking them to correctly identify the content in a 10-second video clip, and one asking them to select a specific option from a list of seven choices; b) when asked to select their religious affiliation, identify as Catholic, Mormon, Protestant, or Christian - Other; c) when asked how religious they are on a 7 pt-scale from 1 (Not at all) to 7 (Extremely), select 4 or greater; and d) report that they had not received any dose of a COVID-19 vaccine. A total of 1,765 participants met this criteria and completed the main dependent variables in the survey. The final sample was 69% female, had a median age of 47, was 83% white, and 32% had a Bachelor’s degree. The distribution of religious affiliation was 22% Catholic, 1.5% Mormon, 38% Protestant, and 38% Christian - Other.

Treatment assignment

We used block randomization based on pretreatment vaccination intentions to assign participants to condition. Before treatment assignment, we asked participants how likely they were to choose to get a COVID-19 vaccine in the next three months, on a scale from 0 to 100. We assigned each to one of four strata: 0, [1-20], [21-75], [76-100]. These strata were determined based on pilot data, in an attempt to include approximately 25% of the sample in each stratum. Within each, participants were randomly assigned to either the control condition or the common religious identity condition.

Analysis

We tested the effect of condition on four variables, each constructed by taking the average of two or more items: vaccination intentions (2 items, alpha = .98), vaccination encouragement (3 items, alpha = .99), shared values with the scientific authority (3 items, alpha = .92), and trust in the scientific authority (2 items, alpha = .95). To model the effect of condition on these outcomes, we regressed each outcome variable on a model that included condition as a dummy variable and controlled for pre-treatment vaccination intentions, gender (as a dummy variable), race (as dummy variables), highest level of education received (as dummy variables), income (continuous), and survey provider (dummy variable). In the models with encouragement as an outcome, we also included a continuous variable with pre-treatment vaccination intentions. To determine significance, we used the beta values associated with the dummy variables for condition, and p-values from two-tailed tests from the regression models.

For moderation analyses, we used the same models as above, but added an additional variable for religiosity (continuous) and interaction between condition and religiosity. We report the beta and p-value for the interaction effect. For statistical mediation analyses, we use Baron and Kenny mediation methods from the *mediation* package in R.

Items

Screening items

Attention checks

Help us keep track of who is paying attention. Please select "Somewhat disagree" in the options below. [Strongly disagree, Disagree, Somewhat disagree, Neither agree nor disagree, Somewhat agree, Agree, Strongly agree]

Please watch the following video with a sound turned on. (Participants view 10-second video of beach and wave sounds).
What did you see in the video? [Saw a beach, heard ocean waves in the background; Saw a beach, heard people speaking in the background; Saw birds, heard ocean waves in the background; Saw birds, heard people speaking in the background]

Received COVID-19 vaccine

Have you received at least one dose of a COVID-19 vaccine? [Yes; No]

Religious affiliation

Which of the following best describes your religious affiliation? [Catholic; Mormon; Protestant-Evangelical; Protestant - Mainline/non-Evangelica; Protestant - Other; Christian - Other; Buddhist; Muslim; Jewish; Athiest; None; Other religion]

Religiosity

How religious are you? [1 (Not at all) - 7 (Extremely)]

Pre-treatment measures

Vaccination Intentions (pre-treatment)

How likely are you to choose to get a COVID-19 vaccine in the next three months? [0 (Extremely unlikely) - 100 (Extremely likely)]

Encouragement Intentions (pre-treatment)

If you had a family member who was unsure whether to get a COVID-19 vaccine or not, how likely would you be to encourage them to get the vaccine? [0 (Extremely unlikely) - 100 (Extremely likely)]

Post-treatment measures

Vaccination Intentions (post-treatment)

How likely are you to choose to get a COVID-19 vaccine? [0 (Never) - 100 (Definitely)]

When, if ever, do you plan to get a COVID-19 vaccine? [0 (Never) - 100 (Immediately)]

Encouragement Intentions (post-treatment)

If you had a family member who was unsure whether to accept a COVID-19 vaccine, how likely would you be to encourage them to get the vaccine? [0 (Extremely unlikely) - 100 (Extremely likely)]

If you had an acquaintance who was unsure whether to accept a COVID-19 vaccine, how likely would you be to encourage them to get the vaccine? [0 (Extremely unlikely) - 100 (Extremely likely)]

If you had a friend who was resistant to getting a COVID-19 vaccine, how likely would you be to encourage them to get the vaccine? [0 (Extremely unlikely) - 100 (Extremely likely)]

Trust in scientific authority

How *trustworthy* did you find the doctor in the video? [0 (Not trustworthy at all) - 100 (Extremely trustworthy)]

How *competent* did you find the doctor in the video? [0 (Not competent at all) - 100 (Extremely competent)]

Shared values with scientific authority

To what extent do you think the doctor in the video shares your values? [0 (Definitely does NOT share my values) - 100 (Definitely does share my values)]

How much do you agree or disagree with the following statement? “The doctor in the video and I care deeply about the same things.” [0 (Strongly disagree) - 100 (Strongly agree)]

How much do you agree or disagree with the following statement? “I believe the doctor in the video is a moral person.” [0 (Strongly disagree) - 100 (Strongly agree)]
